# *tet*-Dependent Gene Expression in Stenotrophomonas maltophilia

**DOI:** 10.1128/spectrum.01576-23

**Published:** 2023-06-28

**Authors:** Rebecca Horch, Diana Rasp, Annika Dietz, Ronald Ebbert, Joerg Steinmann, Ulrich E. Schaible, Uwe Mamat, Ralph Bertram

**Affiliations:** a Institute of Clinical Hygiene, Medical Microbiology and Infectiology, Klinikum Nürnberg, Paracelsus Medical University, Nuremberg, Germany; b Technische Hochschule Nürnberg Georg Simon Ohm, Faculty of Applied Chemistry, Nuremberg, Germany; c Study Program in Human Medicine, Paracelsus Medical University, Nuremberg, Germany; d Department of Cellular Microbiology, Program Area Infections, Research Center Borstel, Leibniz Lung Center, Leibniz Research Alliance INFECTIONS, Borstel, Germany; Forschungszentrum Jülich GmbH

**Keywords:** *Stenotrophomomas maltophilia*, lipopolysaccharide, O antigen, tetracycline, inducible expression, gene regulation

## Abstract

Stenotrophomonas maltophilia is increasingly recognized as an important nosocomial pathogen among the Gram-negative bacteria. Intrinsic resistance to different classes of antibiotics makes treatment of infections challenging. A deeper understanding of S. maltophilia physiology and virulence requires molecular genetic tools. Here, we describe the implementation of tetracycline-dependent gene regulation (*tet* regulation) in this bacterium. The exploited *tet* regulatory sequence of transposon Tn*10* contained the *tetR* gene and three intertwined promoters, one of which was required for regulated expression of a target gene or operon. The episomal *tet* architecture was tested with a *gfp* variant as a quantifiable reporter. Fluorescence intensity was directly correlated with the concentration of the inducer anhydrotetracycline (ATc) applied and the duration of induction. Also, the expression of the *rmlBACD* operon of S. maltophilia K279a was subjected to *tet* control. These genes code for the synthesis of dTDP-l-rhamnose, an activated nucleotide sugar precursor of lipopolysaccharide (LPS) formation. A Δ*rmlBACD* mutant was complemented with a plasmid carrying this operon downstream of the *tet* sequence. In the presence of ATc, the LPS pattern was similar to that of wild-type S. maltophilia, whereas without the inducer, fewer and apparently shorter O-antigen chains were detected. This underscores the functionality and usefulness of the *tet* system for gene regulation and, prospectively, the validation of targets for new anti-S. maltophilia drugs.

**IMPORTANCE** Stenotrophomonas maltophilia is an emerging pathogen in hospital settings and poses a threat to immunocompromised patients. Due to a high level of resistance to different types of antibiotics, treatment options are limited. We here adapted a tool for inducible expression of genes of interest, known as the *tet* system, to S. maltophilia. Genes relevant to producing surface carbohydrate structures (lipopolysaccharide [LPS]) were placed under the control of the *tet* system. In the presence of an inducer, the LPS pattern was similar to that of wild-type S. maltophilia, whereas in the “off” state of the system (without inducer), fewer and apparently shorter versions of LPS were detected. The *tet* system is functional in S. maltophilia and may be helpful to reveal gene-function relationships to gain a deeper understanding of the bacterium’s physiology and virulence.

## OBSERVATION

The Gram-negative bacterium Stenotrophomonas maltophilia is a representative of the *Gammaproteobacteria* and is ubiquitously found in the rhizosphere of plants and other terrestrial and aquatic environments (1). It bears remarkable potential for bioremediation and phytoremediation (2), and furthermore, there is emerging interest in this proficient biofilm former as an opportunistic and nosocomial pathogen (3, 4). Clinical manifestations include infections of numerous anatomic sites (e.g., lung, eyes, heart, gastrointestinal tract, and urinary tract) and bacteremia, with a reported mortality rate of up to 37.5% in immunocompromised patients (5). Treatment of S. maltophilia infections is challenging due to its intrinsic resistance to many antibiotic classes, such as most β-lactams, including carbapenems and cephalosporins, aminoglycosides, and macrolides (3). The virulence repertoire of S. maltophilia includes extracellular factors, such as degradative enzymes of living matter, and cell-associated structures: e.g., pili, adhesins, flagella and lipopolysaccharide (LPS) (6). As a distinctive feature of Gram-negative bacteria, LPS is composed of lipid A, core oligosaccharide, and O-specific polysaccharide, also known as O antigen, frequently found as repetitive units (7). S. maltophilia strains show a pronounced variability in LPS types, reflected in 31 different O serotypes, of which 16 O-antigenic structures have been determined (2, 8).

Molecular tools and techniques for genetic manipulation have paved the way to a deeper understanding of S. maltophilia physiology and virulence. These include transposon mutagenesis, allelic exchange, fluorescent reporters, episomal complementation, and carbohydrate-based gene induction systems (9–12). Here, we applied tetracycline-dependent gene regulation (*tet* regulation) to study the phenotype of an S. maltophilia strain genetically engineered for conditional depletion of an LPS precursor operon.

### A functional *tet* regulation system for S. maltophilia.

**FIG 1:**
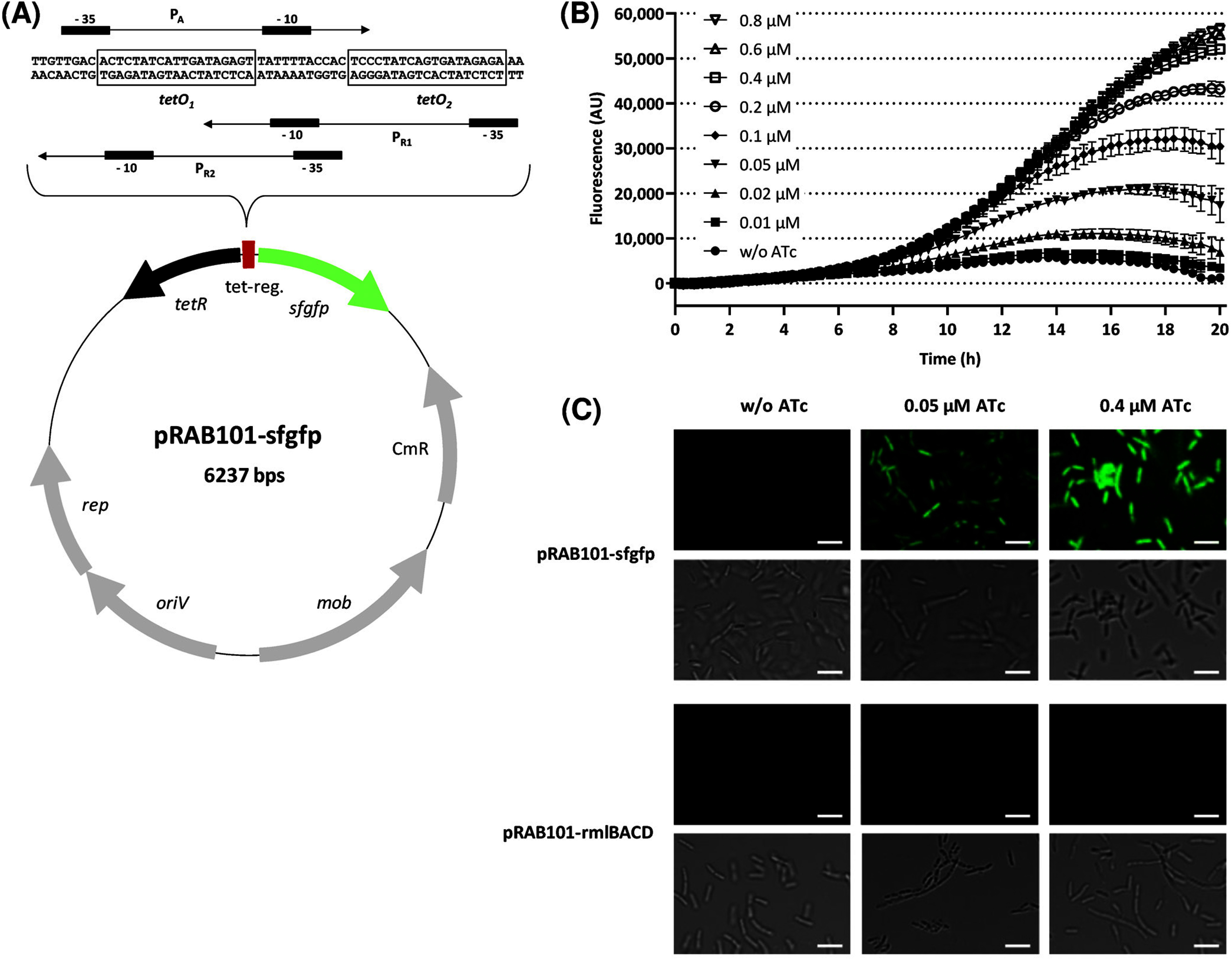
(A) Map of plasmid pRAB101-sfgfp. Features on the plasmid are drawn to scale. The red box indicates the *tet* regulatory sequence, detailed above, with the promoters and P_R1_ and P_R2_ (for expression of *tetR*) and P_A_ (for target gene expression) and the *tet* operators *tetO_1_* and *tetO_2_* marked. (B) Fluorescence measurement of S. maltophilia K279a (pRAB101-sfgfp) in a time-dependent and inducer concentration-dependent manner. Each symbol represents a data point resulting from biological and technical replicates, with standard deviations indicated. The inset describes the ATc concentrations used in each experiment. Corresponding growth curves are provided in Fig. S4. AU, arbitrary units. (C) Microscopy of S. maltophilia K279a bearing pRAB101-sfgfp, or pRAB101-rmlBACD (as a negative control), respectively. Confocal laser scanning results are shown in the upper panels, and corresponding transmitted light images of identical areas are depicted below. Scale bars correspond to 5 μm.

We chose the native Tn*10*-derived *tet* system found in *Enterobacterales* (13) as a new conditional gene regulation unit for S. maltophilia. The system is based upon the tetracycline repressor (TetR) and its cognate DNA sequence *tet* operator (*tetO*). In the absence of inducer, the homodimeric TetR is preferentially bound to *tetO*, which inhibits transcription initiation, whereas its DNA-binding affinity is massively reduced upon interaction with tetracycline or derivatives such as anhydrotetracycline (ATc), leading to target gene induction (14). The *tetR* gene and its upstream region, which harbors two *tetO* sites embedded in three promoters, P_R1_, P_R2_ and P_A_, was amplified from chromosomal DNA of Escherichia coli XL1-Blue (strains summarized in Table S1 in the supplemental material). As a reporter, the superfolder *gfp* gene (*sfgfp*) (15), codon optimized for bacteria of the family *Xanthomonadaceae* (12), was cloned downstream of the P_A_ promoter. These fragments were introduced into the broad-host-range vector pBBR1MCS (16) (plasmids summarized in Table S2) by a modified Gibson assembly protocol (methods detailed in Text S1 in the supplemental material). The resulting plasmid, termed pRAB101-sfgfp (Fig. 1A), was confirmed by sequencing and was introduced into S. maltophilia K279a (17) via triparental mating. Likewise, plasmid pRAB101e, carrying the *tet* region but lacking *sfgfp* (see Fig. S1 in the supplemental material), was used to generate a strain as a negative control. Reporter gene activity of cells grown in liquid culture was determined in a time-dependent and inducer concentration-dependent fashion. Over the time span of 20 h, the fluorescence intensity of S. maltophilia(pRAB101-sfgfp) was directly correlated with the concentration of ATc and increased over the course of time (Fig. 1B). Eight different ATc concentrations were tested, and the response plateaued at about 0.4 μM to 0.8 μM inducer. In the absence of ATc, fluorescence was comparable to that of S. maltophilia(pRAB101e) without *sfgfp* (Fig. S2), indicating tight repression capability. Growth was only weakly affected by either concentration of the inducer tested (Fig. S3 and S4). Confocal laser scanning microscopy was conducted with S. maltophilia(pRAB101-sfgfp) after induction with 0.4 or 0.05 μM ATc for 6 h or without inducer and a likewise-treated negative-control strain containing pRAB101-*rmlBACD* (described below). The latter did not show green fluorescence under any condition tested. Green fluorescence of S. maltophilia(pRAB101-sfgfp) was undetectable in the absence of inducer, whereas upon induction with 0.05 μM ATc, cells glowed dimly green and cells cultivated with 0.4 μM ATc showed bright fluorescence. The response appeared homogeneous, irrespective of the concentration of inducer (Fig. 1C).

### Conditional complementation of the O-antigen precursor operon *rmlBACD*.

**FIG 2:**
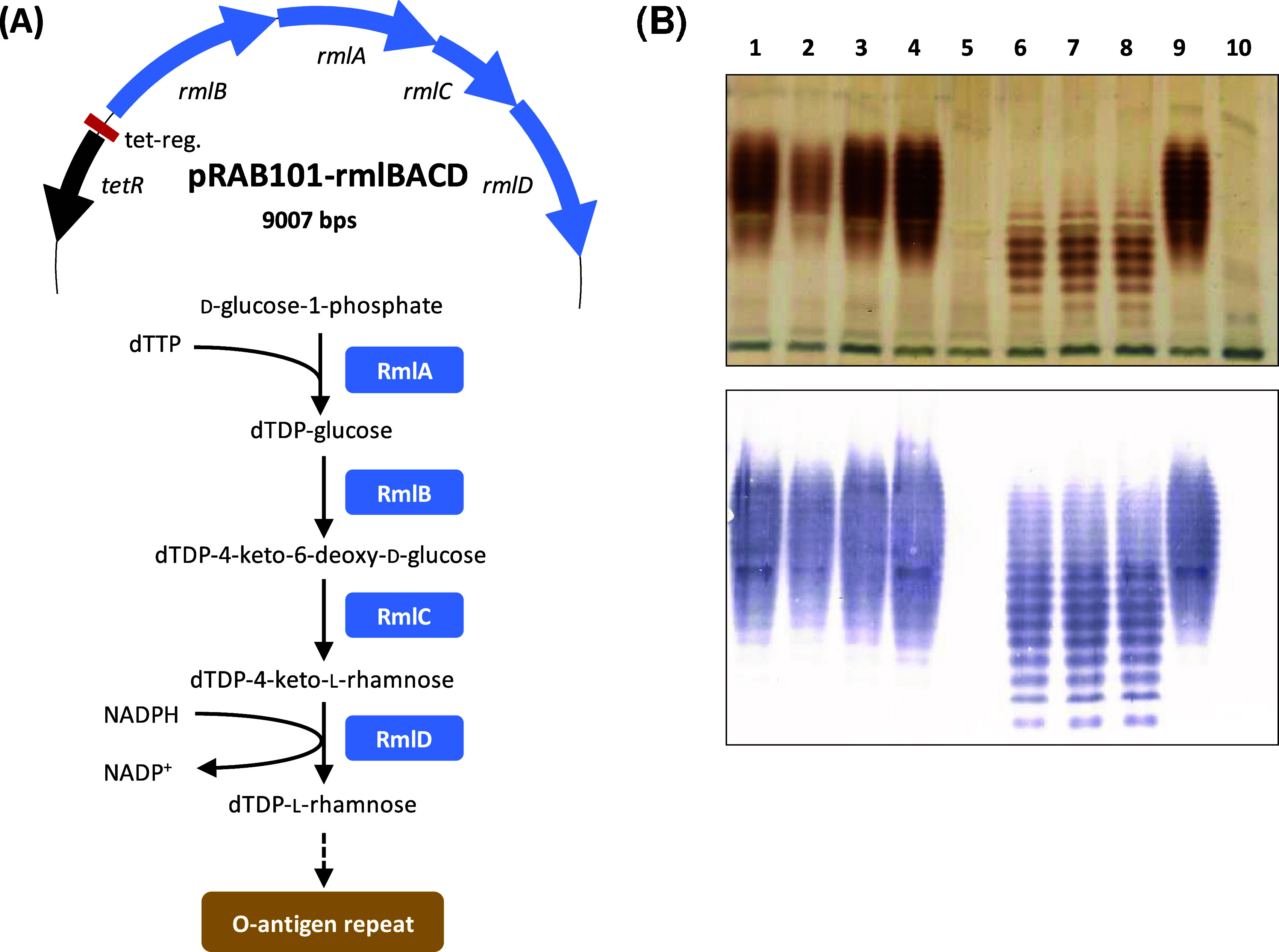
(A, top) Partial map of plasmid pRAB101-rmlBACD. Several features identical to those of pRAB101-sfgfp were omitted; all others are drawn to scale. Below the map are shown schematic steps in the biosynthesis of the rhamnose precursor as part of the O antigen of S. maltophilia K279a. The dashed arrow indicates further steps not resolved in this figure. The actual chemical structure of the O-antigen repeat of S. maltophilia K279a is unknown. (B) SDS-PAGE of LPS preparations, with gels shown after silver staining (top panel) or immunoblotting (bottom panel). Cultures were harvested 6 h after induction. Lanes contained the following samples: lanes 1 to 3 contained three biological replicates of S. maltophilia K279a Δ*rmlBACD*(pRAB101-rmlBACD) grown in the presence of 0.4 μM ATc, lane 4 contained S. maltophilia K279a plus 0.4 μM ATc, lane 5 contained S. maltophilia K279a Δ*rmlBACD* plus 0.4 μM ATc, and lanes 6 to 10 are like lanes 1 to 5, but without ATc.

In order to place native S. maltophilia genes under inducible control, the *tet* regulatory region was cloned upstream of the *rmlBACD* operon of plasmid pBBR1MCS-rmlBACD to obtain pRAB101-rmlBACD (Fig. 2A). The *rml*-encoded enzymes catalyze the conversion of glucose-1-phosphate to dTDP-l-rhamnose, the activated nucleotide sugar precursor for rhamnose residues of the O-antigen repeating unit of K279a-type LPS (2, 8). Despite the occurrence of two nonsynonymous mutations in *rmlA* and one each in *rmlB* and *rmlD* (detailed in the supplemental material), plasmid pRAB101-rmlBACD and also plasmid pRAB101e were transferred to S. maltophilia K279a Δ*rmlBACD* (18) in order to examine conditional complementation capabilities. Cells from cultures of S. maltophilia K279a Δ*rmlBACD*(pRAB101-rmlBACD or pRAB101e) grown in the presence or absence of 0.4 μM ATc for 6 h were treated with proteinase K according to an established protocol for LPS isolation. LPS samples were separated by SDS-PAGE and either silver stained or subjected to immunoblot analysis using an antibody specifically directed against the O-antigenic chain of S. maltophilia K279a (18). As shown in Fig. 2B, the *tet-*repressed state of the plasmid-carried *rml* operon still yielded signals in the complementation strain and thus did not resemble the banding pattern of the *rmlBACD* mutant lacking the O antigen. This indicates leakiness in repression resulting in residual activity of the Rml proteins. However, the banding pattern in the repressed state was clearly different from that of the induced state, which was comparable to that of the S. maltophilia K279a wild type.

### Conclusion.

As shown previously, the difference between the LPS types of S. maltophilia strains is reflected in LPS banding patterns of different lengths (18, 19). It has been noted that mutant strains of S. maltophilia with altered O-antigen repeats show significantly reduced virulence in a rat lung model of infection (20). In a study with E. coli, the repression of a gene critical for LPS synthesis had also resulted in an altered LPS banding pattern, indicative of fewer O-antigen repeats (21). For further studies on the role of LPS in S. maltophilia, inducible expression may be a valuable tool to investigate the virulence potential of resulting strains.

Up to now, two inducible systems had been described for S. maltophilia, both based upon the pBAD promoter, the AraC regulator, and arabinose as an inducer. The first system showed basal leakiness in S. maltophilia and was less tightly repressed than in E. coli (10). The more recent and apparently more efficient setup has been used to regulate the *rmlA* gene (as in the present study), whereas the strain’s phenotype has not been investigated (11). While the pBAD/AraC system is influenced by carbohydrate metabolism, *tet* regulation is rather independent of bacterial physiology and does not require import systems for import of the inducer across the bacterial cell envelope. *tet* regulation has been exploited for gene regulation in more than 40 bacterial genera (22–24). The *tet* regulatory sequence of Tn*10* used in this study is well established for application in at least 11 genera of the alpha-, beta-, and gammaproteobacterial classes (22). Engineered *tet* systems differ in regulatory architecture in the genomic context (e.g., in the promoter sequences) or exploit *tetR* variants with extended or shifted functionalities (22). For future application, the Tn*10*-derived *tet* sequence may be modified to provide tighter repression in S. maltophilia, as shown in other bacteria (25, 26). This could involve exploiting a *tetR* variant with codons adapted to the elevated genomic G+C content typical for S. maltophilia (4). In mycobacteria, codon adaptation has markedly improved *tet* regulation (27). An efficient *tet* system can then mimic gene deletions to provide an even more efficient tool for target validation of future anti-S. maltophilia lead compounds.
