# Supplementary material for: tet-Dependent Gene Expression in Stenotrophomonas maltophilia
Source: Microbiol Spectr. 2023 Jun 28;11(4):e01576-23. doi: 10.1128/spectrum.01576-23 (PMC10434252; doi:10.1128/spectrum.01576-23)
Supplement: Supplemental file 1 — Supplemental material. Download spectrum.01576-23-s0001.docx, DOCX file, 0.03 MB [file spectrum.01576-23-s0001.docx]

**SUPPLEMENTARY MATERIAL**

***Tet*-dependent gene expression** **in *Stenotrophomonas maltophilia***

Rebecca Horch^1,2^, Diana Rasp^3$^, Annika Dietz^2^, Ronald Ebbert^2^, Joerg Steinmann^1^, Ulrich E. Schaible^4^, Uwe Mamat^4^, and Ralph Bertram^1*^

1 Institute of Clinical Hygiene, Medical Microbiology and Infectiology, Klinikum Nürnberg, Paracelsus Medical University, Nuremberg, Germany

2 Technische Hochschule Nürnberg Georg Simon Ohm, Faculty of Applied Chemistry, Nuremberg, Germany

3 Study Program in Human Medicine, Paracelsus Medical University, Nuremberg, Germany

4 Department of Cellular Microbiology, Program Area Infections, Research Center Borstel, Leibniz Lung Center, Leibniz Research Alliance INFECTIONS, Borstel, Germany

$ present address: University Women's Hospital, Klinikum Nürnberg, Paracelsus Medical University, Nuremberg, Germany

ORCID: Ralph Bertram: 0000-0003-0654-6381; Joerg Steinmann: 0000-0002-3181-3667

Contact information of corresponding author

Klinikum Nürnberg

Institute of Clinical Hygiene, Medical Microbiology and Infectiology

Prof.-Ernst-Nathan-Str. 1

90419 Nuremberg, Germany

telephone: +49 911 398 2520

fax: +49 911 398 3266

e-mail: [ralph.bertram@klinikum-nuernberg.de](mailto:ralph.bertram@klinikum-nuernberg.de)

**RUNNING TITLE**

*Tet*-dependent gene expression in *S. maltophilia*

**MATERIALS AND METHODS**

**BACTERIAL STRAINS**

Bacterial strains used in this study are summarized in Table S1.

**PLASMID CONSTRUCTION**

Plasmids used in this study are summarized in Table S2.

pRAB101-sfgfp

A sequence of *sfgfp* (1), codon-optimized for bacteria of the family *Xanthomonadaceae,* was PCR-amplified from plasmid pEX-A2-sfGFP_opt (2)() using primers sfgfp_100_fwd and sfgfp_100_rev (see Table S3 for oligonucleotide sequences). A *tet*-regulation sequence including *tetR* was PCR-amplified from transposon Tn*10* (3) of *E. coli* XL1-Blue (Table S1) using primers Tn10_fwd and Tn10_rev. Plasmid pBBR1MCS-2 (4) was linearized by *Eco*RI digestion and the restriction enzyme was subsequently heat inactivated. About 100 ng of the vector (approx. 0.03 pmol of DNA) were mixed with about 30 ng (approx. 0.06 pmol of DNA) each of the amplified Tn*10 tet*-regulation sequence (758 bp) and the amplified *sfgfp* sequence (831 bp) for an overlap assembly reaction according to the manufacturer’s recommendations (NEBuilder HiFi DNA Assembly Cloning Kit, New England Biolabs (NEB), Frankfurt am Main, Germany). The resulting vector pRAB100 was treated with *Xma*I and *Eco*RV and the 1,545 bp fragment harboring the *tet-sfgfp* sequence was cloned into the likewise digested backbone of plasmid pBBR1MCS (5) to obtain pRAB101 (6,237 bp). This plasmid bears a resistance marker suitable for selection of *S. maltophilia* K279a transformants in Lysogeny Broth (LB) or LB agar with 60 µg/mL of chloramphenicol. All cloning steps were performed using competent *E. coli* NEB5alpha cells (NEB). The *tet-sfgfp* sequence of pRAB101 was verified by Sanger sequencing (GATC, Constance, Germany) and the entire plasmid was sequenced by Oxford Nanopore technology (plasmidsaurus, Eugene, OR, USA). No mutations affecting the *tet*-region, or the reporter gene were observed.

pRAB101e

The *tet*-regulation sequence including *tetR* was PCR-amplified from pRAB101-sfgfp using primers Ptet_fw_101 and tetR_rev_101. The product was digested with *Sac*I and *Eco*RV and cloned into the likewise digested pBBR1MCS backbone to obtain pRAB101e (5,422 bp), see Figure S1.

pBBR1MCS-rmlBACD

For construction of plasmid pBBR1MCS-rmlBACD, the primers 5P-rmlBACD and 3-rmlBACD were designed to PCR-amplify the entire wild-type *rmlBACD* operon (SMLT_RS03085, SMLT_RS03090, SMLT_RS03095, SMLT_RS03100) together with its predicted promoter sequence from the genomic DNA of *S. maltophilia* K279a. The PCR product of 3,645 bp was digested with *Bam*HI/*Hind*III and cloned into the *Bam*HI/*Hind*III sites of the broad host-range vector pBBR1MCS using *E. coli* DH5α as a host bacterium. The *rmlBACD* insert of the final pBBR1MCS-rmlBACD construct of 8,308 bp was verified by sequencing. Reconstitution of O-antigen biosynthesis was confirmed by sodium dodecyl sulfate polyacrylamide gel electrophoresis (SDS-PAGE) following transfer of pBBR1MCS-rmlBACD to *S. maltophilia* K279a Δ*rmlBACD*, as described below.

pRAB101-rmlBACD

The *tet-regulation* sequence including *tetR* was PCR-amplified from plasmid pRAB101 using primers tet_Region_NEB_cloning_fwd and tet_Region_NEB_cloning_rev. Plasmid pBBR1MCS-rmlBACD was linearized by *Pci*I digestion and, after heat inactivation, about 100 ng of the vector (approx. 0.03 pmol) were mixed with about 30 ng (approx. 0.06 pmol) of the pRAB101 *tet*-regulation sequence including *tetR* (738 bp) for an overlap assembly reaction according to the manufacturer’s recommendations (NEBuilder HiFi DNA Assembly Cloning Kit, New England Biolabs, Frankfurt am Main, Germany). The resulting plasmid pRAB101-rmlBACD was subjected to Sanger sequencing (GATC, Constance, Germany) and the entire plasmid was sequenced by Oxford Nanpore technology (plasmidsaurus, Eugene, OR, USA). While the *tet*-regulation sequence including *tetR* was free of mutations, four non-synonymous exchanges in *rml* Genes were detected resulting in the following mutations in the proteins: RmlA T227I and I264V; RmlB I29T; RmlD A68T.

**DELIVERY OF PLASMIDS TO *S. MALTOPHILIA* K279A BY TRIPARENTAL MATING**

Triparental mating was performed as described previously (6, 7). Briefly, *E. coli* DH5α carrying the plasmid pRK2013 (8) served as the helper and *E. coli* NEB5alpha (pRAB101-sfgfp, or pRAB101-rmlBACD, respectively) as the donor for conjugative transfer into the recipient *S. maltophilia* K279a. Transconjugants were selected at 37°C on LB agar plates containing 60 µg/mL chloramphenicol and 5 µg/mL norfloxacin to counter-select against the *E. coli* helper and donor strains. The resulting *S. maltophilia* strains were checked by plasmid preparation and analytical restriction endonuclease digestion.

**REPORTER GENE ASSAY**

A total of 5 µL of overnight cultures of *S. maltophilia* strains of interest were used to inoculate 200 µL of LB medium, supplemented with a final concentration of 60 µg/mL of chloramphenicol and/or anhydrotetracycline (ATc) of various concentrations up to 0.4 µM (approx. 200 ng/mL) in 96-well plate format. Cells were grown at 37°C in a Spark M10 plate reader (TECAN, Männedorf, Switzerland) with shaking for 10 seconds at 200 rpm every 20 minutes, followed by measurement of cell density (OD_600_) and fluorescence (Excitation filter 485 nm, emission filter 535 nm). Measurements were conducted for 20 hours, yielding 60 data points per sample. Each measurement was performed using two biological and three technical replicates thereof.

**CONFOCAL LASER SCANNING MICROSCOPY**

A total of 100 µL of overnight cultures of *S. maltophilia* strains of interest were used to inoculate 5 mL of LB medium supplemented with a final concentration of 60 µg/mL of chloramphenicol and/or ATc of various concentrations up to 0.4 µM. Cells were incubated at 37°C in an orbital shaker at 150 rpm for 6 hours, avoiding direct exposure to light (due to the photo-sensitivity of ATc). Cells were sedimented by centrifugation, resuspended in 20 µL of PBS and mixed with 20 µL of ProLong Live Antifade Reagent (Invitrogen/ThermoFisher) to prevent photobleaching. At total of 10 µL of the suspensions were spotted onto a glass-slide and covered by a cover-slip. Fluorescence microscopy was performed using a Confocal Laser Scanning Microscope SPE-II (Leica Microsystems GmbH, Wetzlar, Germany) with a 63x/1.30 oil objective and LeicaX 3D software. The sfGFP protein was excited with 415-477 nm laser light, and fluorescence emission was detected at 488-561 nm.

**LPS ISOLATION AND DETECTION**

Isolation of LPS and subsequent detection was performed as described previously (9). Briefly, whole-cell lysates of *S. maltophilia* (obtained from cultures, grown in the presence or absence of 0.4 µM ATc for 6 hours,) were treated with proteinase K and then separated by SDS–PAGE. Gels were then either stained with silver nitrate according to the method of Hitchcock and Brown (10) or used for immunoblot analysis. In the latter case, LPS samples were electrotransferred onto polyvinylidene difluoride (PVDF) membranes followed by incubation of the blots with a rabbit polyclonal antibody raised against the O-specific polysaccharide of *S. maltophilia* K279a (9). The membrane was then treated with alkaline phosphatase-conjugated AffiniPure goat anti-rabbit immunoglobulin (Ig)G (HCL) and developed in the presence of nitroblue tetrazolium and 5-bromo-4-chloro-3-indolylphosphate substrate.

**Table S1: Bacterial strains used in this study**

| **Genus and species** | **Strain designation** | **Description/Genotype** | **Reference or source** |
| --- | --- | --- | --- |
| *Escherichia coli* | NEB 5-alpha | fhuA2::IS2 Δ(mmuP-mhpD)169 ΔphoA8 glnX44 ϕ80d[ΔlacZ58(M15)] rfbD1 gyrA96 luxS11 recA1 endA1 rph^WT^ thiE1 hsdR17 | (11)  New England Biolabs (Frankfurt am Main, Germany) |
| *E. coli* | DH5α (pRK2013) | Δ(mmuP-mhpD)169 ΔphoA8 glnX44 ϕ80d[ΔlacZ58(M15)] rfbD1 gyrA96 luxS11 recA1 endA1 rph^WT^ thiE1 hsdR17 and plasmid pRK2013 | (8, 12) |
| *E. coli* | XL1-Blue | *recA1 endA1 gyrA96* *thi-1 hsdR17 supE44 relA1 lac* [F *proAB* *lacIqZ*ΔM15 Tn*10* (Tet^R^)] | Agilent Technologies, Inc. (Santa Clara, CA, USA) |
| *Stenotrophomonas maltophilia* | K279a | Clinical isolate set as wild-type for this study (GenBank accession nr. NC_010943.1) | (13) |
| *S. maltophilia* | K279A Δ*rmlBACD* | Δ*rmlBACD* | (9) |

**Table S2: Plasmids used in this study**

| **Designation** | **Relevant features/Characteristics** | **Reference or source** |
| --- | --- | --- |
| pBBR1MCS | *rep*, *oriV*, *mob*, *cat* (Cm^R^), broad host-range vector | (5) |
| pBBR1MCS-rmlBACD | pBBR1MCS with *rmlBACD* operon from *S. maltophilia* K279a | This study |
| pEX-A2-sfGFP_opt | pEX-A2 with superfolder *gfp* (*sfgfp*), codon adadpted to *Xanthomonadaceae* | (2) |
| pRAB101e | *tetR*, P_A_, P_R1_ and P_R2_ from Tn*10* | This study |
| pRAB101-rmlBACD | pRAB101e with *rmlBACD* | This study |
| pRAB101-sfgfp | pRAB101e with codon-adapted *sfgfp* | This study |

**Table S3: Oligonucleotides used in this study**

| **Designation** | **Sequence (5’ 🡪 3’)** | **Purpose** |
| --- | --- | --- |
| sfgfp_100_fwd | aaaagtgaaagcaggactacatgcg | Amplification of codon-adapted *sfgfp* |
| sfgfp_100_rev | tatcgataagcttgatatcgtggaattgtgagcggataac | Amplification of codon-adapted *sfgfp* |
| Tn10_fwd | tggatcccccgggctgcaggacggaaaaaggttatgctg | Amplification of *tet*-regulatory sequence and *tetR* |
| Tn10_rev | gtcctcctgctttcacttttctctatcactg | Amplification of *tet*-regulatory sequence and *tetR* |
| tetR_rev_101 | attggagctccaccgcggtggc | Amplification of *tet*-regulatory sequence and *tetR* |
| Ptet_fw_101 | ggactggatatcttctctatcactgatagggag | Amplification of *tet*-regulatory sequence and *tetR* |
| 5P-rmlBACD | gagtgggatcccgattctagctggcttgg | Amplification of *rmlBACD* operon. *Bam*HI site for cloning underlined |
| 3-rmlBACD | ctaaagcttgccccgttacctgcgctgtc | Amplification of *rmlBACD* operon. *Hind*III site for cloning underlined |
| tet_Region_  NEB_cloning_fwd | gcagtcgtgaagacgaacgattaagacccactttcacatttaag | Amplification of *tet*-regulatory sequence and *tetR* |
| tet_Region_  NEB_cloning_rev | cggcacagcggcacaggatattctctatcactgataggg | Amplification of *tet*-regulatory sequence and *tetR* |

**LEGENDS TO SUPPLEMENTARY FIGURES**

**Fig. S1:** Map of pRAB101e. Features are drawn to scale. The red box indicates the *tet*-regulatory sequence.

**Fig. S2:** Fluorescence intensity of *S. maltophilia* K279a (pRAB101e) as a function of time and inducer concentration. Each symbol represents a data point resulting from biological and technical replicates, with standard deviations indicated. The inset describes ATc concentrations used in each experiment. Corresponding growth curves are provided in Fig. S3.

**Fig. S3:** Growth curves of *S. maltophilia* K279a (pRAB101e) during the fluorescence measurement (see Fig. S2). Each symbol represents a data point resulting from biological and technical replicates, with standard deviations indicated. The inset describes ATc concentrations used in each experiment.

**Fig. S4:** Growth curves of *S. maltophilia* K279a (pRAB101-sfgfp) in the presence of ATc at different concentrations during the fluorescence measurement (see Fig. 1B). Each symbol represents a data point resulting from biological and technical replicates, with standard deviations indicated. The inset describes ATc concentrations used in each experiment.

**REFERENCES**

1. Pédelacq JD, Cabantous S, Tran T, Terwilliger TC, Waldo GS. 2006. Engineering and characterization of a superfolder green fluorescent protein. *Nat Biotechnol* 24:79-88.

2. Mamat U, Hein M, Grella D, Taylor CS, Scholzen T, Alio I, Streit WR, Huedo P, Coves X, Conchillo-Solé O, Gómez AC, Gibert I, Yero D, Schaible UE. 2023. Improved mini-Tn7 Delivery Plasmids for Fluorescent Labeling of *Stenotrophomonas maltophilia*. *Appl Environ Microbiol*:e0031723.

3. Hillen W, Schollmeier K. 1983. Nucleotide sequence of the Tn*10* encoded tetracycline resistance gene. *Nucleic Acids Res* 11:525-39.

4. Kovach ME, Elzer PH, Hill DS, Robertson GT, Farris MA, Roop RM, Peterson KM. 1995. Four new derivatives of the broad-host-range cloning vector pBBR1MCS, carrying different antibiotic-resistance cassettes. *Gene* 166:175-6.

5. Kovach ME, Phillips RW, Elzer PH, Roop RM, Peterson KM. 1994. pBBR1MCS: a broad-host-range cloning vector. *Biotechniques* 16:800-2.

6. Aubert DF, Hamad MA, Valvano MA. 2014. A markerless deletion method for genetic manipulation of *Burkholderia cenocepacia* and other multidrug-resistant gram-negative bacteria. *Methods Mol Biol* 1197:311-27.

7. Abda EM, Krysciak D, Krohn-Molt I, Mamat U, Schmeisser C, Förstner KU, Schaible UE, Kohl TA, Nieman S, Streit WR. 2015. Phenotypic Heterogeneity Affects *Stenotrophomonas maltophilia* K279a Colony Morphotypes and β-Lactamase Expression. *Front Microbiol* 6:1373.

8. Figurski DH, Helinski DR. 1979. Replication of an origin-containing derivative of plasmid RK2 dependent on a plasmid function provided in trans. *Proc Natl Acad Sci U S A* 76:1648-52.

9. Steinmann J, Mamat U, Abda EM, Kirchhoff L, Streit WR, Schaible UE, Niemann S, Kohl TA. 2018. Analysis of Phylogenetic Variation of *Stenotrophomonas maltophilia* Reveals Huamn-Specific Branches. *Front Microbiol* 9:806.

10. Hitchcock PJ, Brown TM. 1983. Morphological heterogeneity among *Salmonella* lipopolysaccharide chemotypes in silver-stained polyacrylamide gels. *J Bacteriol* 154:269-77.

11. Anton BP, Raleigh EA. 2016. Complete Genome Sequence of NEB 5-alpha, a Derivative of *Escherichia coli* K-12 DH5α. *Genome Announc* 4.

12. Hanahan D. 1983. Studies on transformation of *Escherichia coli* with plasmids. *J Mol Biol* 166:557-80.

13. Avison MB, von Heldreich CJ, Higgins CS, Bennett PM, Walsh TR. 2000. A TEM-2beta-lactamase encoded on an active Tn1-like transposon in the genome of a clinical isolate of *Stenotrophomonas maltophilia*. *J Antimicrob Chemother* 46:879-84.
